# Multicenter Interspecialty Consensus on Experimental Oncology Drug–Related Ocular Adverse Event Reporting

**DOI:** 10.1001/jamaophthalmol.2025.3159

**Published:** 2025-12-04

**Authors:** Neel D. Pasricha, Stella K. Kim, Asim V. Farooq, Ethan S. Lindgren, Rongshan Yan, Gerami D. Seitzman, Matilda F. Chan, Jessica G. Shantha, Dimitra Skondra, Bennie H. Jeng, Winston D. Chamberlain, Kathryn A. Colby, Debra A. Goldstein, Lucia Sobrin, Ivana K. Kim, Kuldev Singh, Wiley A. Chambers, William M. Boyd, Jordyn Silverstein, Paula R. Pohlmann, Janice Lu, Alexa C. Glencer, Laura A. Huppert, A. Jo Chien, Hope S. Rugo, Laura J. Esserman

**Affiliations:** 1 Department of Ophthalmology, University of California, San Francisco; 2 Francis I. Proctor Foundation, University of California, San Francisco; 3 Department of Ophthalmology, University of Texas Health Science Center, Houston; 4 Department of Ophthalmology and Visual Science, University of Chicago Medical Center, Chicago, Illinois; 5 Department of Ophthalmology, Scheie Eye Institute, University of Pennsylvania Perelman School of Medicine, Philadelphia; 6 Casey Eye Institute, Oregon Health & Science University, Portland; 7 Department of Ophthalmology, New York University Grossman School of Medicine, New York University Langone Health, New York; 8 Department of Ophthalmology, Northwestern University Feinberg School of Medicine, Chicago, Illinois; 9 Massachusetts Eye and Ear Infirmary, Harvard Medical School, Boston; 10 Department of Ophthalmology, Byers Eye Institute, Stanford University, Palo Alto, California; 11 Department of Ophthalmology, George Washington University, School of Medicine and Health Sciences, Washington, DC; 12 Division of Ophthalmology, Office of Specialty Medicine, US Food and Drug Administration, Silver Spring, Maryland; 13 Division of Hematology/Oncology, Department of Medicine, David Geffen School of Medicine at the University of California, Los Angeles; 14 Department of Breast Medical Oncology, University of Texas MD Anderson Cancer Center, Houston; 15 Division of Hematology and Oncology, Department of Medicine, Northwestern University Freiberg School of Medicine, Chicago, Illinois; 16 Department of Surgery, University of California, San Francisco; 17 Helen Diller Family Comprehensive Cancer Center, University of California, San Francisco

## Abstract

**Importance:**

The current ocular Common Terminology Criteria for Adverse Events (CTCAE) mix eye signs with symptoms and lack standardized clinical photographs and experimental oncology drug dose modification recommendations. Robust reporting of ocular adverse events (AEs) is important to maintain patient safety and to guide the development of novel efficacious drugs.

**Objective:**

To develop improved ocular AE grading scales to reliably evaluate and grade ocular AEs in patients on experimental oncology drug therapy and to provide clear drug dose modification recommendations.

**Design, Setting, and Participants:**

A collaborative multicenter interspecialty working group consisting of oncologists and academic ophthalmologists from 11 academic centers in the US and ophthalmologists from the US Food and Drug Administration was assembled in February 2023 to form a consensus on new experimental oncology drug–related ocular AE grading scales. The grading scales were released in June 2023.

**Main Outcomes and Measures:**

Expert consensus on novel experimental oncology drug–related ocular AE grading scales.

**Results:**

Six experimental oncology drug–related ocular AE grading scales were developed with agreement from ophthalmologists and oncologists for use in antibody-drug conjugate clinical trials: visual acuity, eye symptoms, cornea, conjunctiva/sclera, anterior chamber, and retina/posterior segment.

**Conclusions and Relevance:**

The new experimental oncology drug–related ocular AE grading scales developed by the consensus panel were developed to be more concise, containing photographs where applicable, and to provide clear drug dose modification recommendations compared with the previous CTCAE. Use of these ocular AE grading scales may allow for more objective and consistent incidence measurements of ocular AEs throughout clinical trials and postmarketing, potentially facilitating safe testing of novel agents that may cause eye toxicity.

## Key Points

**Question:**

Are there possible improvements to the ocular Common Terminology Criteria for Adverse Events (CTCAE)?

**Findings:**

This survey study comprising a consensus panel of ophthalmologists, oncologists, and US Food and Drug Administration personnel found scales ambiguous with unreliable ocular adverse event (AE) grading leading to unclear recommendations for experimental oncology drug dose modifications. New ocular AE grading scales for visual acuity, eye symptoms, cornea, conjunctiva/sclera, anterior chamber, and retina/posterior segment were created with clear grading and associated experimental drug dose modification recommendations.

**Meaning:**

These revised ocular AE grading scales may facilitate consistent reporting, standardize appropriate experimental oncology drug dose modification recommendations, and promote patient safety.

## Teaser

This survey study uses data on ocular adverse events in oncology patients to provide dose modification recommendations.

## Introduction

Antibody-drug conjugates (ADCs) are a class of targeted cancer therapies that consist of a monoclonal antibody linked to a cytotoxic payload by a chemical linker. Since 2000, with the first US Food and Drug Administration (FDA)–approved ADC, gemtuzumab ozogamicin for acute myeloid leukemia, there are currently 12 FDA-approved ADCs.^1,2^ ADCs are now widely studied across tumor types and more than 160 ADCs are currently in clinical trials, including in I-SPY 2 (NCT01042379), an adaptive clinical trial platform testing experimental drugs in the neoadjuvant setting for patients with high-risk early-stage breast cancer.^3,4^

An ocular adverse event (AE) associated with ADCs is corneal pseudomicrocysts, also known as microcystlike epithelial changes, leading to blurred vision and eye pain in up to 90% of patients on select ADC therapy, making this the most common AE associated with several ADCs.^5,6^ Ocular preventive therapies have demonstrated limited efficacy. Fortunately, the corneal pseudomicrocysts appear reversible with modification of ADC therapy, including dose delay, dose reduction, and drug discontinuation.^6^ Since visual function and ocular health play major roles in determining patient quality of life and work productivity, accurately grading ADC drug—related ocular AEs along with timely communication between eye care clinicians and oncologists is important.^7^

The National Cancer Institute Common Terminology Criteria for Adverse Events (CTCAE) provides grading scales for reporting AEs, including ocular AEs such as blurred vision, eye pain, and keratitis, and is the gold standard for toxicity assessment in oncology trials.^8^ Use of the most updated CTCAE version 5.0 to grade ocular AEs from ADCs in the I-SPY 2 trial led to the identification of at least 4 limitations:

1. Ambiguous CTCAE terms (eg, *dry eye* and *keratitis*).
2. Mixed signs and symptoms (eg, keratitis grades include visual acuity, objective clinical examination findings, and subjective patient symptoms).
3. No representative clinical photographs.
4. No experimental oncology drug dose modification recommendations.

These limitations posed a challenge for curable patients enrolled in the I-SPY 2 trial who were receiving ADC therapy and experiencing corneal pseudomicrocysts; eye care clinicians were unable to accurately grade their ocular AEs and oncologists were unsure if ADC dose modification was necessary. Oncologists are often uncomfortable with acute ocular conditions; inconsistent classification of ocular AEs, along with fear of vision loss, raised serious concerns about the use of new ADCs in the curative early-stage high-risk setting. Clinical protocols initially required patients with grade 2 keratitis to hold ADC therapy until keratitis resolution. However, there was no consensus on what constituted grade 2 keratitis, and complete resolution of keratitis was not clearly defined. This led to more than 50% of I-SPY 2 patients receiving select ADC therapies to prematurely discontinue treatment without a clear need to do so. The long time to resolution of keratitis required ADC discontinuation, eliminating the ability to effectively test ADC therapy in these patients. Importantly, there was no long-term ocular morbidity from these cases of grade 2 keratitis. A prompt regulatory strategy was needed to minimize ocular risk while continuing to safely offer high-risk breast cancer patients access to potentially effective systemic ADC therapy. This report describes a case study of the FDA Center of Excellence in Regulatory Science and Innovation (CERSI) sponsoring a multicenter interspecialty consensus working group on revising ADC-related ocular AE reporting, which ultimately could be applied to many experimental oncology drug–related ocular AEs, including non-ADCs.

## Methods

This study was exempt from institutional review board approval given that no patient data were collected and member responses were deidentified. The Strengthening the Reporting of Observational Studies in Epidemiology (STROBE) reporting guideline was followed. In February 2023, a multicenter interspecialty consensus working group on oncology drug–related ocular AEs was formed on February 12, 2023, and consisted of 23 leading ophthalmologists and oncologists from 11 academic centers in the US involved in the I-SPY 2 trial across the US and the FDA. Consensus was achieved using a nominal group technique. Members were tasked with reviewing the literature and drawing on their knowledge of oncology drug–related AEs to form a consensus on new ocular AE grading scales. Efforts were focused on using unambiguous terms, separating signs and symptoms, using representative clinical photographs when indicated, and providing clear experimental drug dose modification recommendations. Multiple virtual meetings were held with each member having the opportunity to review the data and comment on the collective working group recommendations.

### Statistical Analysis

The consensus working group members were surveyed in deidentified fashion (Qualtrics) for their 5-point Likert agreement score with each of the 6 new ocular AE grading scales individually, overall and in comparison to the ocular CTCAE version 5.0. The η^2^ effect size from the *t* tests of the 2 groups was calculated using GraphPad Prism version 10 (GraphPad Software).

## Results

Six novel experimental oncology drug–related ocular AE grading scales were created by the multicenter interspecialty consensus working group. Ambiguous umbrella terms such as *dry eye* were replaced with unambiguous anatomical terms like *cornea* that no longer mixed signs and symptoms. When appropriate, representative clinical photographs were used to illustrate ocular AE grades. Each ocular AE grade included an associated ADC dose modification recommendation. Scales run from grade 0 to 4. The updated ocular AE grading scales were implemented into the I-SPY 2 trial protocol on June 22, 2023. Recognizing the possible utility of the updated ocular AE grading scales for experimental oncology drugs beyond ADCs, the consensus working group adapted the ocular AE grading scales into its current form (Table; eAppendix in Supplement 1).

**Table.** Experimental Oncology Drug–Related Ocular Adverse Events Grading Scales

Grade	Eye symptoms^a^	Visual acuity	Cornea	Conjunctiva/sclera	Anterior chamber	Retina/posterior segment	Drug recommendation
0	No eye symptoms (0/10)	Baseline VA 20/20 Snellen equivalent VA (if no baseline VA)^b^	Clear	White and quiet Trace hyperemia	Quiet	Normal	Continue
1	Mild eye symptoms (1-3/10)	1-Line VA decrease 20/25 Snellen equivalent VA (if no baseline VA)^b^	Nonconfluent epitheliopathy	Mild (1-≥2) hyperemia	Trace ≥0.5 cells (1-5 cells)^c^	Trace ≥0.5 vitreous cells (1-5 cells)^d^	Continue with close ocular follow-up
2	Moderate eye symptoms (4-6/10)	2-3–Line VA decrease 20/30-20/40 Snellen equivalent VA (if no baseline VA)^b^	Confluent epitheliopathy	Moderate-severe (3-≥4) hyperemia Episcleritis Conjunctival epithelial defect Membranous conjunctivitis Subepithelial fibrosis	1-≥2 Cells (6-25 cells)^c^ 1-≥2 Flare (clear iris details)	≥1 Vitreous cells (6-10 cells)^d^ Trace-≥1 vitreous haze (mild blurring of optic nerve details) Intraretinal or subretinal fluid	Cornea: continue with close ocular follow-up Noncornea: delay dose until Grade 1^e^, then consider resuming full dose or reduced dosing
3	Severe eye symptoms (7-9/10)	4-8–Line VA decrease but better than 20/200 Snellen equivalent VA 20/50-20/150 Snellen equivalent VA (if no baseline VA)^b^	Epithelial defect Corneal ulcer	Scleritis Symblepharon or forniceal shortening Ocular surface keratinization	3-≥4 Cells (≥26 cells)^c^ 3-≥4 Flare (hazy iris details, fibrin or plasmoid aqueous) Hypopyon	2-≥3 Vitreous cells (11-50 cells)^d^ 2-≥3 Vitreous haze (moderate-marked blurring of optic nerve details) Retinal or vitreous hemorrhage, choroidal detachment, retinal or choroidal neovascularization, retinal vascular abnormalities, retinal break, retinal or choroidal lesion Nonmacular retinal detachment	Delay dose until grade 1^e^, then consider reduced dosing or discontinuation
4	Extreme eye symptoms (10/10)	>8-Line VA decrease or 20/200 Snellen equivalent or worse VA	Corneal perforation	Ankyloblepharon Scleral necrosis or perforation	NA	≥4 Vitreous cells (>50 cells)^d^ ≥4 Vitreous haze (no optic nerve details) Macular-involving retinal detachment	Consider discontinuation

Abbreviations: NA, not applicable; VA, visual acuity (measured as best-corrected visual acuity or pinhole-corrected visual acuity).

^a^ Eye symptoms include eye pain, eye discomfort, tearing, light sensitivity, and blurry vision at time of questioning without topical ocular anesthesia.

^b^ Use decrease in VA unless no baseline VA is available.

^c^ High-intensity 1 × 1-mm slit beam.

^d^ High-intensity 1 × 0.5-mm slit beam.

^e^ Or until stable for chronic nonprogressive structural findings.

### Eye Symptoms

Eye symptoms AE grades (eAppendix in Supplement 1) evaluate eye pain, eye discomfort, tearing, light sensitivity, and blurry vision (subjective) at the time of patient questioning without applying topical ocular anesthesia. The Wong-Baker FACES Pain Rating Scale, which includes 6 cartoon faces and is the gold standard for pain assessment, is included to facilitate patient scoring on a 0 to 10 scale.^9^

### Visual Acuity

Visual acuity AE grades (eAppendix in Supplement 1) are based on definitive loss of Snellen equivalent best-corrected visual acuity or pinhole-corrected visual acuity.^10,11^ Given some patients may have decreased visual acuity at baseline due to conditions unrelated to experimental oncology drug therapy (eg, age-related macular degeneration), the visual acuity AE grades are based on change from baseline visual acuity, when a baseline visual acuity measurement is available.

### Cornea and Conjunctiva/Sclera

Cornea AE grades (eAppendix in Supplement 1) and conjunctiva/sclera AE grades (eAppendix in Supplement 1) are based on slitlamp examination or anterior segment eye imaging with and without fluorescein staining using cobalt blue light. Of note, corneal pseudomicrocysts are not included in the cornea AE grades since they may be difficult to visualize even with high magnification slitlamp examination and up to 30% of patients with these lesions are asymptomatic.^5^ Advancing from nonconfluent (grade 1) to confluent (grade 2) epitheliopathy is important to document for toxicity progression but both can be managed medically with close ocular follow-up. For conjunctiva/sclera AE grades, the validated Efron conjunctival hyperemia (redness) scale and representative artist-rendered photographs were used.^12^

### Anterior Chamber

Anterior chamber AE grades (eAppendix in Supplement 1) are based on a modification of the Standardization of Uveitis Nomenclature criteria, which require high magnification slitlamp examination.^13^

### Retina/Posterior Segment

Retina/posterior segment AE grades (eAppendix in Supplement 1) are the most extensive of the 6 ocular AE grading scales due to the complexity and variety of clinical findings. While the representative photographs only cover vitreous haze, both retina and vitreous findings are covered in the text.^14^ For vitreous cells, the Multicenter Uveitis Steroid Treatment trial criteria were used, which requires high magnification slitlamp examination.^15^

The interspecialty multicenter consensus group showed excellent agreement for each of the 6 new drug–related ocular AE grading scales (mean [SEM]: visual acuity,4.5 [0.1]; eye symptoms, 4.3 [0.2], cornea, 4.3 [0.2], conjunctiva/sclera, 4.3 [0.2]; anterior chamber, 4.3 [0.1]; retina/posterior segment, 4.3 [0.1]), overall (4.4 [0.1]), and in comparison to the ocular CTCAE version 5.0 (4.6 [0.1]) among the ophthalmologists and oncologists (Figure; eAppendix in Supplement). The overall η^2^ effect size was 0.003.

**Figure.**
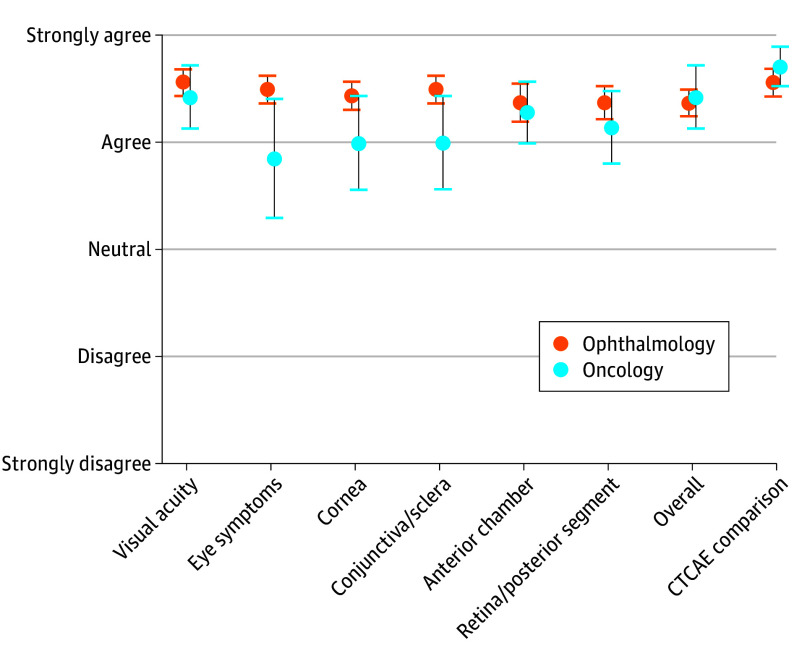
Consensus Group Agreement There are 23 individuals represented in the figure data, 16 in ophthalmology and 7 in oncology. Data are presented as means (SEMs) on a 5-point Likert agreement scale.

## Discussion

This survey study demonstrates prompt implementation of regulatory change to promote patient safety while continuing to support the advancement of innovative therapeutics. A CERSI-sponsored multicenter interspecialty group of ophthalmologists and oncologists was able to efficiently reach initial consensus on novel ADC drug–related ocular AE grading scales within a few months. The draft ocular AE grading scales were successfully implemented into the I-SPY 2 trial, which includes ADCs under investigation in the neoadjuvant setting for patients with high-risk early-stage breast cancer. While the current study was inspired by ADC-related corneal toxicity (corneal pseudomicrocysts), the 6 updated ocular AE grading scales may be applicable for other experimental oncology drug–related ocular AEs. Further deliberation and inclusion of additional specialists facilitated the consensus development of the present version of ocular AE grading scales, which has the potential for broad use across other experimental oncology drug–related ocular AEs. For experimental oncology drugs that use the new consensus ocular AE grading scales, these scales can continue to be used postmarketing. Ultimately, the goal is use of the consensus ocular AE grading scales for all oncologic drug–related AEs. Compared to the CTCAE version 5.0, the new ocular AE grading scales provide 4 main improvements:

1. Clear anatomical AE terms (eg, *cornea* and *conjunctiva/sclera*).
2. Separate signs and symptoms (eg, visual acuity grades for vision changes and cornea grades for objective corneal clinical findings).
3. Representative clinical photographs, if applicable.
4. Experimental oncology drug dose modification recommendations.

Importantly, the new ocular AE grading scales are meant to be used together such that signs and symptoms are graded independently and the dosing decision is based on the highest grade. Although the consensus group had excellent agreement on the new ocular AE grading scales overall, as evidenced by a low η^2^ value, 2 important clarifications were discussed. First, experimental oncology drug dose modification recommendations are provided to help prevent vision loss or irreversible damage to the eyes, but the final dosing decision remains at the discretion of the treatment team. This is important to allow for medical decision-making in unique clinical scenarios. For example, if there are multiple reasonable alternative therapies available to a patient, drug discontinuation may be warranted even for grade 1 ocular AEs. Conversely, if a patient is receiving life-extending therapy with no effective alternatives, drug may be continued despite grade 3 or 4 ocular AEs. Second, the new ocular AE grading scales are intended for experimental oncology drugs; approved drugs may potentially have used different ocular AE grading scales, such as the CTCAE. Thus, the new consensus ocular AE grading scales are not intended to replace the CTCAE version 5.0; however, future CTCAE versions may be updated to align with this grading scheme.

These ocular AE grading scales will facilitate reliable grading by eye care clinicians and clear experimental oncology drug dose modification recommendations to oncologists. Effective communication between eye care clinicians and oncologists is crucial for patients experiencing ocular AEs receiving experimental oncology drug therapy, including ADC therapy, since ADC-induced corneal pseudomicrocysts appear to be reversible with ADC dose modifications. We used a nominal group technique consensus approach to reach agreement and were able to efficiently implement new ocular AE grading scales. To date, to our knowledge, dose modifications proposed by the new ocular AE grading scales in the I-SPY 2 trial have not caused any irreversible eye toxicity. We are studying alternative methods to screen for ocular AEs in patients, such as portable screening with eye imaging in the oncology clinic. Additionally, we are exploring the underlying mechanisms of ADC-induced corneal toxicity and the development of novel preventive therapies.

### Limitations

This study has limitations. The report could be strengthened by collecting data on ocular AE grading scale usability, such as real-world surveys from clinical use. Since the new ocular AE grading scales were initially designed for use with ADCs, we realize certain theoretical ocular AEs are not included, such as elevated intraocular pressure, cataract, ptosis, or periocular changes. If relevant for future experimental oncology drugs, additional ocular AE grading scales may be added. While dose modification recommendations are provided, standardization is challenging due to the different mechanisms of action across different agents.

## Conclusions

The CERSI-sponsored multicenter interspecialty working group of ophthalmologists and oncologists reached consensus on 6 new experimental oncology drug–related ocular AE grading scales. An initial draft of updated ocular AE grading scales was efficiently implemented into the I-SPY 2 trial protocol, providing high-risk breast cancer patients better-informed access to neoadjuvant ADC therapy, which has the known ocular AE of corneal pseudomicrocysts associated with blurred vision and eye pain. Confusion over how to classify ocular AEs and how to manage them could have stopped the evaluation of ADCs in the early-stage setting. Accurate classification and dosing guidance through revised ocular AE scales resolved these issues and allowed for safe evaluation of promising ADC drugs. Compared to CTCAE, the new ocular AE grading scales contain fewer ambiguous terms, separate signs and symptoms, provide representative clinical photographs if applicable, and clearly state experimental oncology drug dose modification recommendations. We propose use of the nomenclature *consensus ocular AE grading scales* to differentiate from previous systems. These new ocular AE grading scales have facilitated safe and effective evaluation of eye toxicity from ADCs in the curable breast cancer setting. This concerted multidisciplinary effort is a model for addressing emerging toxicities associated with new therapies, providing clarity and consistent grading for AE reporting. The CERSI mechanism continues to provide an optimal communication channel with regulatory authorities to rapidly solve a significant clinical issue in clinical trial management.

## Notes

Supplement 1. New Experimental Oncology Drug-Related Ocular Adverse Events Grading Scales

Supplement 2. Data sharing statement
