# Supplementary material for: Multicenter Interspecialty Consensus on Experimental Oncology Drug–Related Ocular Adverse Event Reporting
Source: JAMA Ophthalmol. 2025 Dec 4;144(1):62–8. doi: 10.1001/jamaophthalmol.2025.3159 (PMC12679423; doi:10.1001/jamaophthalmol.2025.3159)
Supplement: Supplement 1. — New Experimental Oncology Drug-Related Ocular Adverse Events Grading Scales [file jamaophthalmol-e253159-s001.pdf]

# Supplemental Online Content

Pasricha ND, Kim SK, Farooq AV, et al. Multicenter interspecialty consensus on experimental oncology drug-related ocular adverse event reporting. *JAMA Ophthalmol*. Published online December 4, 2025. doi:10.1001/jamaophthalmol.2025.3159

**eAppendix.** New experimental oncology drug-related ocular adverse events grading scales

This supplemental material has been provided by the authors to give readers additional information about their work.

© 2025 Pasricha ND et al. JAMA Ophthalmology.

**eAppendix.** New Experimental Oncology Drug-Related Ocular Adverse Events Grading Scales

**A)** Eye Symptoms Adverse Events Grading Scale

**B)** Visual Acuity Adverse Events Grading Scale

When measuring pinhole-corrected visual acuity (PCVA), patients wear their prescription distance vision spectacles, if applicable, and hold a pinhole occluder in front of one eye at a time. Unlike best-corrected visual acuity (BCVA), PCVA is an efficient way to correct most refractive error while avoiding the need to perform time-consuming refraction required for BCVA.[13] The simple Snellen chart was chosen instead of the more complex Early Treatment Diabetic Retinopathy Study (ETDRS) chart since many eye care providers, let alone oncologists, are unfamiliar with ETDRS letter scoring or do not have access to ETDRS charts in their clinics.[14] A Snellen-equivalent VA conversion chart was included to accommodate international VA notation standards. In cases where the patient did not have an eye exam prior to initiating experimental oncology drug therapy and no baseline VA is available, the VA value can be used.

**C)** Cornea Adverse Events Grading Scale

Grade 1 (non-confluent) is distinguished from grade 2 (confluent) epitheliopathy given confluent epitheliopathy may signal worsening corneal disease. However, epitheliopathy may be managed while continuing experimental oncology drug therapy with close ocular follow-up. Grade 3 distinguishes between epithelial defect (grade 3A) where the corneal epithelium is absent but the underlying stroma is intact and corneal ulcer (grade 3B) where there is stromal involvement.

**D)** Conjunctiva/Sclera Adverse Events Grading Scale

The cicatricial structural findings included in grades 2 and 3 of the Conjunctiva/Sclera AEs (e.g. subepithelial fibrosis, symblepharon or forniceal shortening, and ocular surface keratinization) are not expected to resolve to grade 1. Therefore, the experimental oncology drug therapy recommendations in these cases are modified to delay until the structural findings are stable. This allows for patients to dose delay and then potentially resume their experimental oncology drug at a reduced dose following stabilization (e.g. stable palpebral conjunctival subepithelial fibrosis).

© 2025 Pasricha ND et al. JAMA Ophthalmology.

**E)** Anterior Chamber Adverse Events Grading Scale

Despite being the gold standard in grading anterior uveitis for decades, the Standardization of Uveitis Nomenclature (SUN) criteria do not have representative clinical photos. Of note, there is no grade 4 Anterior Chamber AE given significant anterior chamber inflammation, including a hypopyon, may be reasonably managed with dose delay and reduction for some patients without long-term ocular sequalae (e.g. reactive hypopyon from a corneal epithelial defect).

**F)** Retina/Posterior Segment Adverse Events Grading Scale

Notably, the Multicenter Uveitis Steroid Treatment (MUST) trial specifies a 1 x 0.5 mm slit beam instead of the 1 x 1 mm slit beam indicated for the Standardization of Uveitis Nomenclature (SUN) criteria. For vitreous haze, the scale and representative fundus photos created by Nussenblatt et al. and affirmed by the SUN working group were used.[11,15] Intraretinal or subretinal fluid (e.g. central serous retinopathy) is grade 2 given its reversibility. As with the Conjunctiva/Sclera AE grading scale, the structural findings included in grade 3 (e.g. retinal or choroidal lesion) are not always expected to resolve to grade 1. Therefore, the experimental oncology drug therapy recommendation in these cases is modified to delay until the structural findings are stable. This allows for patients to dose delay and then potentially resume their experimental oncology drug at a reduced dose following stabilization (e.g. stable peripheral chorioretinal scar).

© 2025 Pasricha ND et al. JAMA Ophthalmology.

# EYE SYMPTOMS

| CLINICAL FINDINGS | | DRUG RECOMMENDATION |
|---|---|---|
| **GRADE 0**<br>No eye symptoms (0/10) | 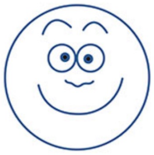<br>0 | Continue |
| **GRADE 1**<br>Mild eye symptoms (1-3/10) | 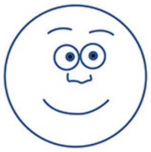<br>2 | Continue with close ocular follow-up |
| **GRADE 2**<br>Moderate eye symptoms (4-6/10) | 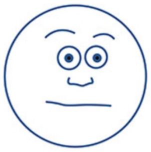 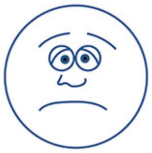<br>4 6 | Delay dose until Grade 1, then consider resuming full dose or reduced dosing |
| **GRADE 3**<br>Severe eye symptoms (7-9/10) | 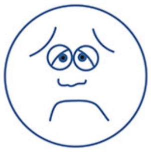<br>8 | Delay dose until Grade 1, then consider reduced dosing or discontinuation |
| **GRADE 4**<br>Extreme eye symptoms (10/10) | 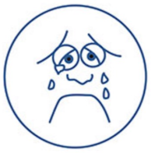<br>10 | Consider discontinuation |

Includes eye pain, eye discomfort, tearing, light sensitivity, and/or blurry vision at time of questioning without topical ocular anesthesia


References:

- Wong-Baker FACES Foundation (2022). Wong-Baker FACES® Pain Rating Scale. Retrieved August 27, 2025 with permission from http://www.WongBakerFACES.org. Originally published in *Whaley & Wong's Nursing Care of Infants and Children*. © Elsevier Inc.


***DRUG RECOMMENDATIONS ARE AT THE DISCRETION OF THE TREATMENT TEAM***

© 2025 Pasricha ND et al. JAMA Ophthalmology.

# VISUAL ACUITY

| CLINICAL FINDINGS | DRUG RECOMMENDATION |
|---|---|
| **GRADE 0**<br>Baseline VA<br>20/20 Snellen equivalent VA (if no baseline VA)* | Continue |
| **GRADE 1**<br>1–line VA decrease<br>20/25 Snellen equivalent VA (if no baseline VA)* | Continue with close ocular follow-up |
| **GRADE 2**<br>2-3–line VA decrease<br>20/30-20/40 Snellen equivalent VA (if no baseline VA)* | Delay dose until Grade 1, then consider resuming full dose or reduced dosing |
| **GRADE 3**<br>4-8–line VA decrease but better than 20/200 Snellen equivalent VA<br>20/50-20/150 Snellen equivalent VA (if no baseline VA)* | Delay dose until Grade 1, then consider reduced dosing or discontinuation |
| **GRADE 4**<br>>8–line VA decrease or 20/200 Snellen equivalent or worse VA | Consider discontinuation |

VA: Best-corrected visual acuity (BCVA) or pinhole corrected visual acuity (PCVA)

*Use VA decrease unless no baseline VA is available

References:
- Reprinted with permission from the American Academy of Ophthalmology. Visual Acuity Conversion Chart from BCSC 2020-2021 series: Section 3 - Clinical Optics

Table 3-2 **Visual Acuity Conversion Chart**

| Snellen Fraction | | | | | |
|---|---|---|---|---|---|
| Feet | Meters | 4-Meter Standard | Decimal Notation (Visus) | Visual Angle Minute of Arc | LogMAR (Minimum Angle of Resolution) |
| 20/10 | 6/3 | 4/2 | 2.00 | 0.50 | –0.30 |
| 20/15 | 6/4.5 | 4/3 | 1.33 | 0.75 | –0.12 |
| **20/20** | **6/6** | **4/4** | **1.00** | **1.00** | **0.00** |
| 20/25 | 6/7.5 | 4/5 | 0.80 | 1.25 | 0.10 |
| 20/30 | 6/9 | 4/6 | 0.67 | 1.50 | 0.18 |
| 20/40 | 6/12 | 4/8 | 0.50 | 2.00 | 0.30 |
| 20/50 | 6/15 | 4/10 | 0.40 | 2.50 | 0.40 |
| 20/60 | 6/18 | 4/12 | 0.33 | 3.00 | 0.48 |
| 20/80 | 6/24 | 4/16 | 0.25 | 4.00 | 0.60 |
| 20/100 | 6/30 | 4/20 | 0.20 | 5.00 | 0.70 |
| 20/120 | 6/36 | 4/24 | 0.17 | 6.00 | 0.78 |
| 20/150 | 6/45 | 4/30 | 0.13 | 7.50 | 0.88 |
| 20/200 | 6/60 | 4/40 | 0.10 | 10.00 | 1.00 |
| 20/400 | 6/120 | 4/80 | 0.05 | 20.00 | 1.30 |

*DRUG RECOMMENDATIONS ARE AT THE DISCRETION OF THE TREATMENT TEAM*

© 2025 Pasricha ND et al. JAMA Ophthalmology.

# CORNEA

CLINICAL FINDINGS | DRUG RECOMMENDATION

**GRADE 0**

Clear

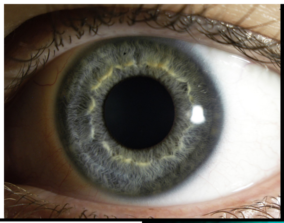
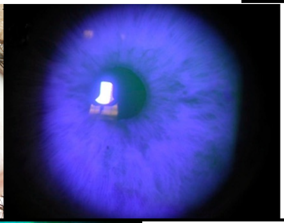

Continue

---

**GRADE 1**

Non-confluent epitheliopathy

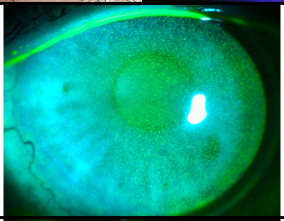

Continue with close ocular follow-up

---

**GRADE 2**

Confluent epitheliopathy

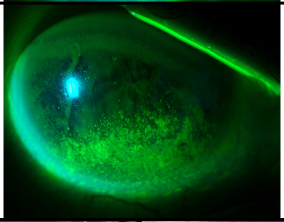

Continue with close ocular follow-up

---

**GRADE 3**

**A**: Epithelial defect
**B**: Corneal ulcer

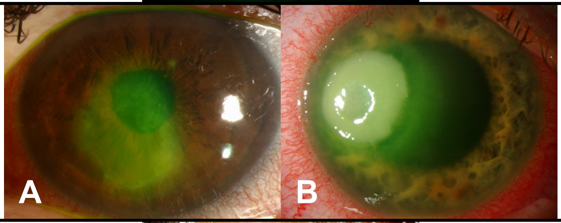


Delay dose until Grade 1, then consider reduced dosing or discontinuation

---

**GRADE 4**

Corneal perforation

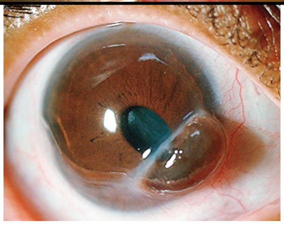

Consider discontinuation

References:
- Clear Cornea #1 (left): Wiessner E. Iris cornea adnexa - biomicroscope. Wikimedia Commons. https://commons.wikimedia.org/wiki/File:Iris_cornea_adnexa_-_biomicroscope.jpg. Published June 11, 2014
- Clear Cornea #2 (right): Reprinted with permission from Chan EH et al. Epithelial Thinning in Limbal Stem Cell Deficiency. *Am J Ophthalmol*. 2015;160(4):669-77.e4
- Non-Confluent Epitheliopathy: Vislisel J. Punctate epithelial erosions. EyeRounds.org, The University of Iowa. https://webeye.ophth.uiowa.edu/eyeforum/atlas/pages/punctate-epithelial-erosions-3/index.htm
- Confluent Epitheliopathy: Vislisel J. Punctate epithelial erosions. EyeRounds.org, The University of Iowa. https://webeye.ophth.uiowa.edu/eyeforum/atlas/pages/Punctate-epithelial-erosions/index.htm
- Epithelial Defect: Vislisel J. Corneal abrasion. EyeRounds.org, The University of Iowa. https://webeye.ophth.uiowa.edu/eyeforum/atlas/pages/corneal-abrasion/index.htm
- Corneal Ulcer: Chen JJ. Pseudomonas keratitis. EyeRounds.org, The University of Iowa. https://webeye.ophth.uiowa.edu/eyeforum/atlas/pages/pseudomonas-keratitis-34.html
- Corneal Perforation: Reprinted with permission from Nataneli N et al. Images in clinical medicine: Bilateral corneal perforation. N Engl J Med. 2014;370(7):650

***DRUG RECOMMENDATIONS ARE AT THE DISCRETION OF THE TREATMENT TEAM***

© 2025 Pasricha ND et al. JAMA Ophthalmology.

# CONJUNCTIVA/SCLERA

CLINICAL FINDINGS | DRUG RECOMMENDATION

## GRADE 0

White and quiet
Trace hyperemia

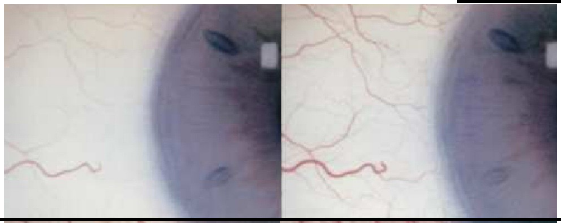

Continue

## GRADE 1

Mild (1-2+) hyperemia

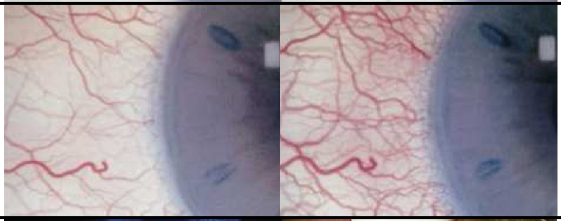

Continue with close ocular follow-up

## GRADE 2

Moderate-severe (3-4+) hyperemia
Episcleritis
Conjunctival epithelial defect
Membranous conjunctivitis
Subepithelial fibrosis

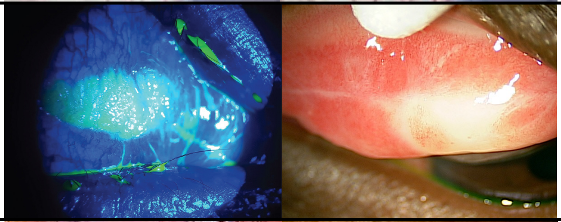

Delay dose until Grade 1†, then consider resuming full dose, reduced dosing, or discontinuation

## GRADE 3

Scleritis
Symblepharon or forniceal shortening
Ocular surface keratinization

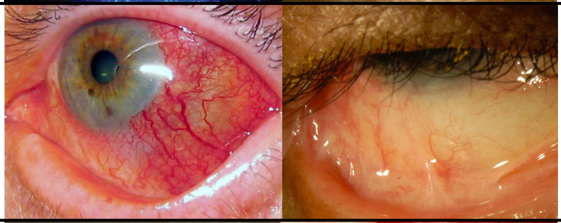

Delay dose until Grade 1†, then consider reduced dosing or discontinuation

## GRADE 4

Ankyloblepharon
Scleral necrosis or perforation

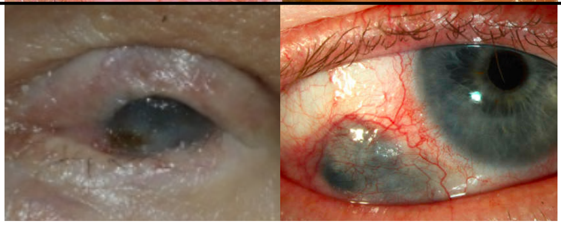

Consider discontinuation

†Or until stable for chronic, non-progressive structural findings

References:

- Conjunctival Hyperemia: Reprinted with permission from Sánchez et al. On the development of conjunctival hyperemia computer-assisted diagnosis tools. *Artif Intell Med*. 2016;71:30-42
- Conjunctival Epithelial Defect: Raju et al. Hair Shaft in the Lacrimal Punctum: A Rare Presentation. *TNOA Journal of Ophthalmic Science and Research.* 2022;60(2):212-213
- Conjunctival Subepithelial Fibrosis: Courtesy of Asim Farooq, MD (University of Chicago Medicine)
- Scleritis: Doan A. Scleritis. EyeRounds.org, The University of Iowa. https://webeye.ophth.uiowa.edu/eyeforum/atlas/pages/scleritis.html#gsc.tab=0
- Symblepharon: Reprinted with permission from Khan R. Symblepharon formation in patient with OCP. EyeWiki. https://eyewiki.org/File:Symblepharon_formation_in_patient_with_OCP.png. Published November 30, 2015
- Ankyloblepharon: Reprinted with permission from Auteri et al. Ocular Cicatricial Pemphigoid: summary of pathogenesis, diagnosis & treatment. *Moran CORE*. https://morancore.utah.edu/section-08-external-disease-and-cornea/ocular-cicatricial-pemphigoid-summary-of-pathogenesis-diagnosis-treatment/
- Scleral Necrosis: Courtesy of Gerami Seitzman, MD (Francis I. Proctor Foundation, University of California San Francisco)

***DRUG RECOMMENDATIONS ARE AT THE DISCRETION OF THE TREATMENT TEAM***

© 2025 Pasricha ND et al. JAMA Ophthalmology.

# ANTERIOR CHAMBER

| CLINICAL FINDINGS | DRUG RECOMMENDATION |
| --- | --- |
| **GRADE 0**<br>Quiet | Continue |
| **GRADE 1**<br>Trace 0.5+ cells (1-5 cells)‡ | Continue with close ocular follow-up |
| **GRADE 2**<br>1-2+ cells (6-25 cells)‡<br>1-2+ flare (clear iris details) | Delay dose until Grade 1, then consider resuming full dose or reduced dosing |
| **GRADE 3**<br>3-4+ cells (≥26 cells)‡<br>3-4+ flare (hazy iris details, fibrin or plasmoid aqueous)<br>Hypopyon | Delay dose until Grade 1, then consider reduced dosing or discontinuation |

‡High-intensity 1 x 1 mm slit beam

References:

- Standardization of Uveitis Nomenclature (SUN) Working Group. Standardization of uveitis nomenclature for reporting clinical data. *Am J Ophthalmol*. 2005;140(3):509-16

***DRUG RECOMMENDATIONS ARE AT THE DISCRETION OF THE TREATMENT TEAM***

© 2025 Pasricha ND et al. JAMA Ophthalmology.

# RETINA/POSTERIOR SEGMENT

CLINICAL FINDINGS — DRUG RECOMMENDATION

## GRADE 0

Normal

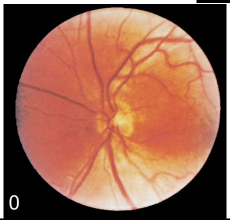


Continue

## GRADE 1

Trace 0.5+ vitreous cells (1-5 cells)§

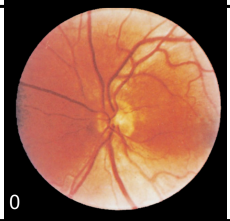


Continue with close ocular follow-up

## GRADE 2

1+ vitreous cells (6-10 cells)§
Trace-1+ vitreous haze (mild blurring of optic nerve details)
Intraretinal or subretinal fluid

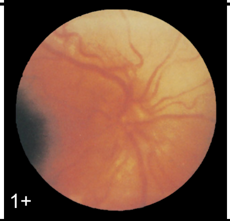


Delay dose until Grade 1, then consider resuming full dose, reduced dosing, or discontinuation

## GRADE 3

2-3+ vitreous cells (11-50 cells)§
2-3+ vitreous haze (moderate-marked blurring of optic nerve details)
Retinal or vitreous hemorrhage, choroidal detachment, retinal or choroidal neovascularization, retinal vascular abnormalities, retinal break, retinal or choroidal lesion
Non-macular retinal detachment

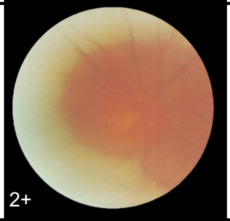


Delay dose until Grade 1†, then consider reduced dosing or discontinuation

## GRADE 4

4+ vitreous cells (>50 cells)§
4+ vitreous haze (no optic nerve details)
Macular-involving retinal detachment

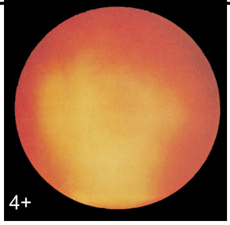


Consider discontinuation

†Or until stable for chronic, non-progressive structural findings

§High-intensity 1 x 0.5 mm slit beam

References:
- MUST Trial Research Group. The multicenter uveitis steroid treatment trial: rationale, design, and baseline characteristics. *Am J Ophthalmol*. 2010;149(4):550-561
- Reprinted with permission from Nussenblatt et al. Standardization of vitreal inflammatory activity in intermediate and posterior uveitis. *Ophthalmology*. 1985;92(4):467–471

***DRUG RECOMMENDATIONS ARE AT THE DISCRETION OF THE TREATMENT TEAM***

© 2025 Pasricha ND et al. JAMA Ophthalmology.
