# Supplementary material for: Multicenter Interspecialty Consensus on Experimental Oncology Drug–Related Ocular Adverse Event Reporting
Source: JAMA Ophthalmol. 2025 Dec 4;144(1):62–8. doi: 10.1001/jamaophthalmol.2025.3159 (PMC12679423; doi:10.1001/jamaophthalmol.2025.3159)
Supplement: Supplement 2. — Data sharing statement [file jamaophthalmol-e253159-s002.pdf]

Data Sharing Statement

Pasricha. Multicenter Interspecialty Consensus on Experimental Oncology Drug–Related Ocular Adverse Event Reporting. *JAMA Ophthalmol*. Published December 04, 2025. doi:10.1001/jamaophthalmol.2025.3159

Data
Data available: No

Additional Information
Explanation for why data not available: N/A
